# Ocular metastasis from esophageal cancer: a systematic review and pooled analysis of individual-level case data

**DOI:** 10.1007/s00595-026-03373-2

**Published:** 2026-07-13

**Authors:** Adam Mylonakis, Marisophie P. Vasilakopoulou, Markos Despotidis, Aristeidis Sourgiadakis, Dimitra Stefanou, Petros C. Petrou, Eirini Chatziralli, Dimitrios Schizas

**Affiliations:** 1 First Department of Surgery, National and Kapodistrian University of Athens, Laikon General Hospital, 17 Agiou Thoma Str, Athens, 11527 Greece; 2 Department of Ophthalmology, General Hospital of Chalkida, Chalkida, Greece; 3 First Department of Medicine, National and Kapodistrian University of Athens, Laikon General Hospital, Athens, Greece; 4 First Department of Opthalmology, National and Kapodistrian University of Athens, “G. Gennimatas” Hospital, Athens, Greece; 5 Second Department of Opthalmology, National and Kapodistrian University of Athens, Attikon University Hospital, Athens, Greece

**Keywords:** Choroidal Neoplasms, Eye Neoplasms, Secondary, Neoplasm Metastasis, Visual Acuity, Radiotherapy, External Beam.

## Abstract

**Purpose:**

Ocular metastasis from esophageal cancer is a very rare clinical entity, typically only described in isolated case reports. This systematic review describes the clinical features, diagnostic strategies, treatment modalities, and outcomes of this uncommon manifestation.

**Methods:**

A systematic search of the literature in the PubMed/Medline and Scopus databases was conducted from database inception to January 25, 2026, focusing on adult ocular metastasis from esophageal cancer.

**Results:**

Forty-one cases were identified (mean age 58.7 ± 10.5 years; male-to-female ratio 5.8:1). The most common presenting symptom was blurred vision (46.3%), followed by eye pain (26.8%), diplopia and ptosis (each, 14.6%), and floaters (9.8%). Impaired visual acuity was documented in 85.3% of the cases reported. The choroid was the most frequently involved site (43.8%), followed by the iris (31.3%) and then the retina (12.5%). Ocular metastasis was synchronous in 63.4% of cases and was the presenting manifestation in 29.3%. Additional systemic metastases were present in 80.5% of cases. External beam radiotherapy was the most common local treatment (68.3%), achieving visual improvement in approximately two-thirds of treated patients. Median overall survival was 6 months.

**Conclusions:**

Ocular metastasis from esophageal cancer is indicative of advanced systemic disease. Early recognition and prompt local therapy may preserve visual function and quality of life despite poor overall prognosis.

## Introduction

Esophageal cancer, one of the most lethal malignancies worldwide, is characterized by its aggressive behavior and poor prognosis. With an estimated 511,000 new cases in 2022, it contributed to approximately 445,000 cancer-related deaths worldwide in the same year [1]. Despite advances in diagnosis and multimodal therapy, esophageal cancer is frequently diagnosed at an advanced stage, and is associated with poor long-term survival [2, 3].

Common metastatic sites of esophageal carcinoma include the liver, lungs, bones, and lymph nodes, reflecting its propensity for both hematogenous and lymphatic spread [4, 5]. In contrast, ocular metastasis from esophageal cancer has rarely been described in the literature, often limited to isolated case reports and small case series [6, 7]. When ocular metastases occur, the choroid is the most frequently affected site, because of its rich vascular supply [8]. Other reported locations include the iris, retina, vitreous, extraocular muscles, and orbit. The proposed mechanism for ocular dissemination involves hematogenous spread through the posterior ciliary arteries, with tumor cells lodging in the choroidal vasculature or adjacent structures [9]. Given the immune-privileged nature of the eye and its anatomical barriers, these metastases are rare and typically signal widespread systemic dissemination [10]. Owing to their rarity and nonspecific symptoms, ocular metastases are often misdiagnosed or identified at an advanced stage. Clinical presentations vary and may include blurred vision, floaters, diplopia, eye pain, or even cranial nerve palsy. In certain cases, ocular involvement may precede diagnosis of the primary esophageal malignancy, highlighting the need for clinician awareness. We analyzed published cases of esophageal cancer metastasizing to ocular structures, focusing on the demographic characteristics, clinical presentation, timing and laterality of metastasis, diagnostic methods, treatment modalities (systemic and local), and patient outcomes. By synthesizing the existing literature, this review aims to promote clinical recognition of this rare phenomenon and guide diagnostic and therapeutic strategies in affected patients.

## Materials and methods

This systematic review and pooled analysis of individual-level case data was conducted following the Preferred Reporting Items for Systematic Reviews and Meta-Analyses (PRISMA) guidelines and registered in the PROSPERO database (ID: CRD420251041099) [11]. Two investigators searched the PubMed/MEDLINE and Scopus databases independently, from database inception to January 25, 2026 to identify reports of esophageal cancer with ocular metastasis, using the following search algorithm: (“esophageal“[All Fields] OR “esophagus“[All Fields]) AND (“metastasis“[All Fields] OR “ocular“[All Fields] OR “eye“[All Fields] OR “choroid“[All Fields] OR “retina“[All Fields] OR “orbit“[All Fields] OR “uvea“[All Fields] OR “iris“[All Fields]). Any controversies were adjudicated at the discretion of a senior investigator. Articles written in English, presenting case reports or case series were included in this systematic review, whereas reviews and meta-analyses were excluded. Articles in which the diagnosis was not clearly established and articles with insufficient clinical data were also excluded. Data extraction of the pertinent studies was performed independently by two authors using a predefined and pre-established form. Information regarding gender, age, ocular symptomatology, and cancer history were compiled. Additionally, data were gathered on the timing and laterality of metastasis, diagnostic modalities, anatomical site of ocular involvement, treatment approaches (systemic and local), and clinical outcomes.

Numerical variables are presented as means ± standard deviation (SD), whereas categorical variables are described as frequencies and valid percentages. Patients included in case series were considered as unique cases to estimate the variables of interest. Survival analysis was performed using the Kaplan–Meier method. Because of the lack of required information in several studies, rates were calculated based on the available data. Statistical analysis was conducted using IBM SPSS Statistics for Windows, Version 29.0 (IBM Corp, NY).

## Results

**Fig. 1:**
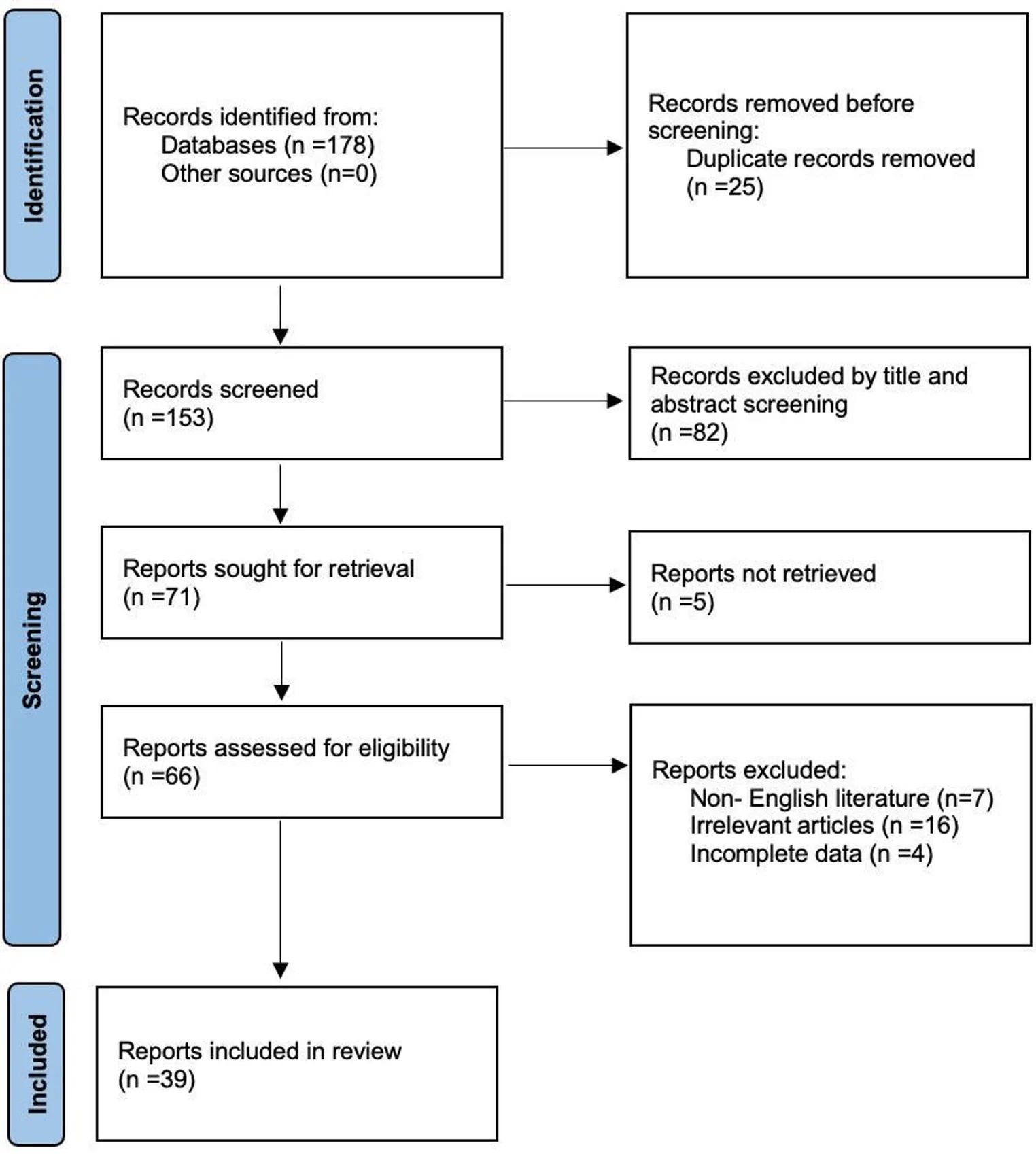
Trial flowchart of this review

This meta-analysis includes 39 studies, reporting on a collective total of 41 patients with esophageal metastasis to the eye. Fig. 1 presents a detailed trial flowchart. There were 38 individual case reports and 1 case series. Six (14.6%) of the 41 patients were women and 35 were men (85.4%), yielding a male-to-female ratio of 5.8:1. The mean age at presentation was 58.7 ± 10.5 years. Histological data were available for 38 of the 41 patients. The most frequently reported histological subtype was adenocarcinoma, diagnosed in 23 patients (60.5%). Squamous cell carcinoma was identified in 14 (36.8%) patients, while a single case (2.6%) was attributed to primary esophageal melanoma [12–50].

Synchronous ocular metastasis, defined as occurring at or within 6 months of the primary cancer diagnosis, was diagnosed in 26 patients (63.4%). In 12 of these patients (29.3%), ocular symptoms were the initial manifestation that led to the diagnosis of esophageal cancer. The remaining 15 patients (36.6%) had metachronous metastasis, with ocular involvement developing later. For these metachronous presentations, the interval between primary cancer diagnosis and ocular metastasis ranged from 12 to 84 months (mean ± SD: 29 ± 22 months). Thirteen of these 15 patients (86.7%) had undergone esophagectomy and 13 (86.7%) had received systemic chemotherapy, with regimens including cisplatin plus 5-fluorouracil (5-FU), carboplatin plus paclitaxel, cisplatin plus irinotecan, and epirubicin-based combinations. Nine patients (60%) were treated with radiation therapy, either preoperatively or postoperatively, with doses ranging from 45 to 70.2 Gy. Only two patients (13.3%) did not receive structured oncologic treatment before ocular metastasis onset. Table 1 summarizes the patients’ demographic characteristics.

**Table 1:** Clinical, histological, and metastasis characteristics of the cases of ocular metastasis from esophageal cancer reported in the literature

Variable	Result
Age mean (range)	58.7 (41-83)
Gender	
Male	35 (85.4%)
Female	6 (14.6%)
Histological Type Reported (%)	38 (100%)
Adenocarcinoma	23(60.5%)
Squamous Cell Carcinoma	14 (36.8%)
Melanoma	1 (2.6%)
Metastasis Diagnosis Timing	
Synchronous ocular metastasis	26 (63.4%)
Metachronous ocular metastasis	15 (36.6%)
Metachronous Disease	
Mean interval from primary diagnosis (range)	29 (12-84) months
Previous Esophagectomy	13 (86.7%)
Previous Chemotherapy	13 (86.7%)
Previous Radiation therapy	9 (60%)

Blurred vision was the most frequently reported symptom, observed in 19 patients (46.3%), although visual acuity data at presentation were available for 34 patients. Specifically, impaired vision was observed in 29 patients (85.3%), while 5 (14.7%) maintained normal visual acuity. Eye pain was reported for 11 patients (26.8%), followed by diplopia, ptosis and swelling/edema, each reported for 6 patients (14.6%). Other symptoms included floaters, visual field defects, redness, proptosis and headache (in 4 patients each; 9.8%). Less frequent manifestations were photopsia, metamorphopsia, cranial nerve palsy, hyphema, and photophobia, and one patient was asymptomatic. Most patients had combinations of symptoms, reflecting varied anatomical involvement.

The location of ocular metastasis was reported in 32 cases. The most affected site was the choroid (*n* = 14; 43.8%), followed by the iris (*n* = 10; 31.3%), retina (*n* = 4; 12.5%), extraocular muscles (*n* = 2; 6.3%), vitreous (*n* = 1; 3.1%), and sclera (*n* = 1; 3.1%). Laterality was documented in 39 cases, with unilateral involvement in 32 (82.1%) and bilateral involvement in 7 (17.9%). Table 2 shows the tumor locations and clinical presentations of each patient. Analyzing the clinical characteristics of ocular metastasis, eye examination findings reflected a broad spectrum of intraocular and orbital involvement. The most commonly affected site was the choroid (43.8%), with patients presenting elevated yellow-white masses frequently associated with exudative or serous retinal detachment, subretinal fluid, and retinal pigment epithelium changes. There were several cases of bilateral choroidal lesions or macular involvement. Iris involvement, observed in 31.3% of patients, was characterized by visible vascularized masses, neovascularization, stromal thickening, hyphema, posterior synechiae, and elevated intraocular pressure. There were cases of angle closure and ciliary body extension. Vitreous infiltration typically presented as vitritis, white clumps, or veil-like opacities. Retinal abnormalities, including tractional detachment, white infiltrative lesions, and intraretinal hemorrhages, were reported in approximately 24.4% of patients. Anterior segment signs ranged from shallow anterior chambers and flare to cataract formation. Orbital involvement was seen in 17.1% of patients, manifesting as proptosis, extraocular muscle infiltration, and as cranial nerve III palsy. The ocular lesion was externally visible as a scleral or iris nodule in approximately 5% of patients.

**Table 2:** Location and clinical presentation in patients with ocular esophageal metastasis reported in the literature

Variable	Result
Ocular Metastasis Location Reported	32 (100%)
Choroid	14 (43.8%)
Iris	10 (31.3%)
Retina	4 (12.5%)
Extraocular muscles	2 (6.3%)
Vitreous	1 (3.1%)
Sclera	1 (3.1%)
Metastastis Laterality Reported	39 (100%)
Unilateral Metastasis	32 (82.1%)
Bilateral Metastasis	7 (17.9%)
Visual Acuity Reported	34 (100%)
Impaired Vision	29 (85.3%)
Normal Vision	5 (14.7%)
Clinical Presentation	
Blurred Vision	19 (46.3%)
Eye Pain	11 (26.8%)
Diplopia	6 (14.6%)
Ptosis	6 (14.6%)
Swelling/Edema	6 (14.6%)
Floaters	4 (9.8%)
Visual field defects (scotoma, peripheral loss)	4 (9.8%)
Redness/Hyperemia	4 (9.8%)
Proptosis	4 (9.8%)
Headache	4 (9.8%)
Photopsia	2 (4.9%)
Metamorphosia	2 (4.9%)
Cranial nerve palsy	2 (4.9%)
Hyphema	1 (2.4%)
Photophobia	1 (2.4%)
Asymptomatic	1 (2.4%)

Percentages may exceed 100% as patients could present with multiple symptoms

A broad range of diagnostic modalities was employed to evaluate ocular involvement and confirm metastatic disease. Fundoscopic examination served as the primary ophthalmic assessment tool and was performed in 35 of the 41 patients (85.4%). Optical Coherence Tomography (OCT) and Magnetic Resonance Imaging (MRI) were utilized in 30 cases (73.2%), while B-scan ultrasonography and Computed Tomography (CT) were used in 20 cases each (48.8%). Positron emission tomography-computed tomography (PET-CT) was performed in 18 patients (43.9%), slit-lamp examination was performed in 12 (29.3%) and fluorescein angiography was performed in 10 (24.4%), particularly for anterior and posterior segment evaluation. Tissue diagnosis was obtained through fine-needle aspiration biopsy in 6 patients (14.6%), orbitotomy in 6 (14.6%), vitrectomy in 5 (12.2%), and iris or transretinal biopsy in 4 (9.8%). Systemic work-up included esophageal biopsy in 18 patients (43.9%) and upper gastrointestinal endoscopy in 15 (36.6%). Histopathology and immunohistochemical staining were performed in 13 patients (31.7%) to confirm the diagnosis and identify the esophageal origin of the ocular lesion.

The eye was the only site of metastatic disease at the time of evaluation in eight patients (19.5%), potentially representing an isolated metastatic presentation. In contrast, systemic metastatic disease beyond the eye was reported in 33 patients (80.5%). The most commonly involved site was the liver, in 18 patients (43.9%), followed by lymph nodes in 15 patients (36.6%) (including mediastinal, abdominal, supraclavicular, cervical, and para-aortic regions), and the lung in 13 patients (31.7%). Bone metastases were identified in 10 patients (24.4%), involving the vertebrae, ribs, pelvis, femur, and other skeletal locations. Brain metastases were reported in 7 patients (17.1%), including cerebral and subcortical lesions; pleural involvement was reported in 3 patients (7.3%); and adrenal metastases were reported in 2 patients (4.9%). Other less frequent metastatic sites included the skin and subcutaneous tissue, pericardium, pancreas, spleen, and retroperitoneum.

Systemic therapy was administered to 31 of the 41 patients (75.6%) with esophageal cancer and ocular metastasis, but 10 (24.4%) did not receive systemic treatment following ocular involvement. The most common regimens included platinum-based doublets, fluoropyrimidine-based combinations, and taxane-containing protocols. A subset of patients also received immunotherapy, including PD-1 inhibitors, particularly for progressive or refractory disease. Targeted therapies, such as HER2-directed agents, were given to a limited number of patients with confirmed molecular markers [26, 28]. Systemic therapy was not given to five patients for reasons such as advanced clinical deterioration, treatment refusal, exhaustion of prior therapies, or transition to best supportive care. Three patients had already been treated with systemic therapy before the onset of ocular metastasis, and no further treatment was initiated. For two other patients, systemic therapy had been planned but was not given because of rapid clinical deterioration [17, 19].

Several local therapeutic approaches were used to manage ocular metastases, with external beam radiotherapy (EBRT) being the most frequent. EBRT was delivered in several regimens, including conventional fractionation (such as 40 Gy in 20 fractions or 60 Gy in 30 fractions) and Stereotactic Body Radiotherapy (SBRT) (such as 35–40 Gy over 14–21 fractions). Overall, 28 out of 41 patients (68.3%) received eye-directed radiotherapy and 2 of these patients also received adjunctive intraocular therapies as intravitreal injections of bevacizumab or triamcinolone [23, 26]. Eight patients underwent surgery, primarily for diagnostic or palliative purposes. Refractory symptoms such as intractable pain or secondary glaucoma necessitated enucleation in two patients [17, 19]. Less invasive surgical procedures were performed in three patients, including pars plana vitrectomy for diagnostic sampling or retinal detachment repair, and excision of scleral or orbital lesions [13, 18, 27]. Table 3 summarizes the treatment modalities reported.

**Table 3:** Summary of systemic and local treatment modalities reported in patients with ocular metastasis from esophageal cancer

Treatment Category	Patients *n* (%)
Systemic therapy	
Any systemic therapy administered	31 (75.6%)
Platinum-based chemotherapy regimens	24 (58.5%)
Immunotherapy (PD-1 inhibitors)	3 (7.3%)
Targeted therapy (HER2-directed agents)	2 (4.9%)
Not Specified	2 (4.9%)
No systemic therapy after ocular metastasis	10 (24.4%)
Local ocular therapy	
Radiotherapy to ocular/orbital structures	26 (63.4%)
Radiotherapy to ocular/orbital structures + Intravitreal therapy (bevacizumab ± triamcinolone)	2 (4.9%)
Surgical treatment	8 (19.5)
Vitrectomy / pars plana vitrectomy	3 (7.3%)
Surgical excision of orbital/scleral lesion	3 (7.3%)
Enucleation	2 (4.9%)
Observation / no local ocular therapy	5 (12.1%)

PD-1, programmed death-1; HER2, human epidermal growth factor receptor 2

**Fig. 2:**
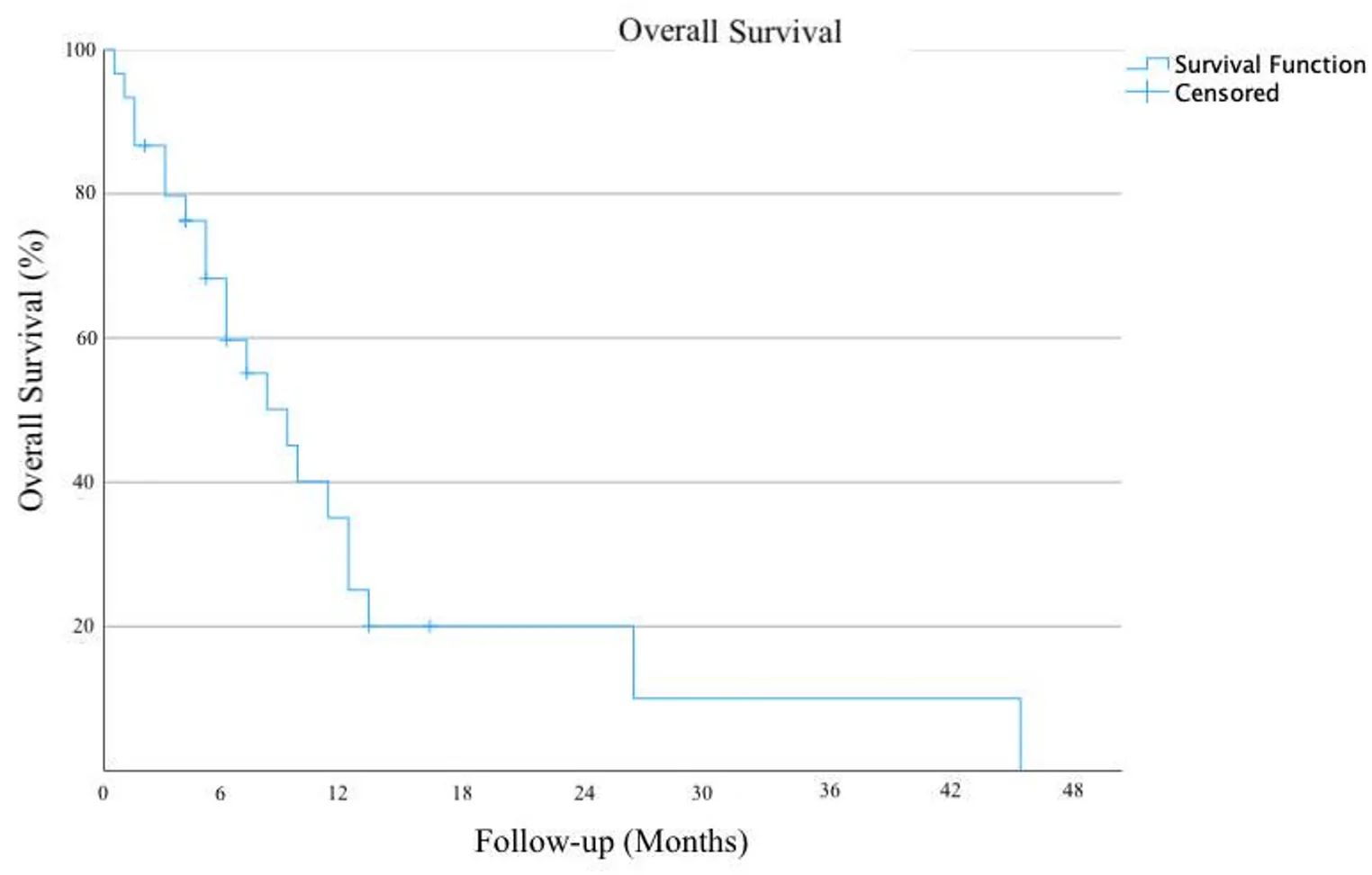
Cumulative survival Kaplan-Meier curve of patients with ocular esophageal metastasis

Visual outcome data following local ocular therapy were available for 12 patients. Clinical improvement was reported for eight of these patients (66.7%), either in visual acuity or in resolution of ocular symptoms and four (33.3%) had stable ocular disease without further visual deterioration. Improvements were most frequently noted in patients treated with radiotherapy, with symptom resolution including blurred vision, floaters, photopsia, or diplopia, and improvement of visual acuity to baseline or near-baseline levels. Follow-up data were available for 30 patients. The follow-up period ranged from 2 weeks to 45 months, with a median duration of 16.9 months. The median survival for the cohort was 6 months, whereas 1-year and 2-year survival rates were 23.7% (95% CI: 6%–41.4%) and 9.5% (95% CI: 0%–25%), respectively. Fig. 2 shows the cumulative survival. Univariate Cox regression analysis revealed that none of the evaluated clinical or disease-related variables, including gender, age, visual acuity, laterality, timing of metastasis, or extent of systemic involvement, were significantly associated with overall survival (Table 4).

**Table 4:** Unifactorial Cox proportional analysis regarding overall survival of patients with ocular esophageal metastasis

	UNIFACTORIAL ANALYSIS				
	HR	95% CI			*p*-value
Female gender	1.831	0.505	-	6.636	0.357
Age >60 years	0.957	0.62	-	1.748	0.940
Impaired Visual Acuity	0.554	0.156	-	1.963	0.360
Bilateral Metastasis	1.953	0.700	-	5.446	0.201
Metachronous Metastasis	0.943	0.389		2.289	0.897
Ocular-only Metastasis	2.27	0.305	-	17.07	0.424
Liver involvment	1.753	0.561	-	5.471	0.334
Brain involvment	1.695	0.557	-	5.152	0.352

## Discussion

Ocular metastasis from esophageal carcinoma is an exceptionally rare clinical entity, reported only in case reports and small case series. Esophageal cancer typically spreads via the lymphatic and hematogenous route to regional and distant lymph nodes and visceral organs. In advanced esophageal carcinoma, over 50% of esophageal metastasis involve the liver, followed by distant lymph nodes, lung, peritoneum, bone, and brain [3, 4]. The mechanism of metastasis of esophageal carcinoma to ocular structures is not fully understood, but is presumed to involve hematogenous dissemination via the rich vascular supply of the choroid, particularly the posterior ciliary arteries .

The eye as a metastatic site stands in contrast to the common metastatic pathways of esophageal cancer. In the largest reported series of ocular metastases, the most common primary tumors originated from the breast (37%) and lung (27%), followed by kidney (4%), gastrointestinal tract (4%), cutaneous melanoma (2%), lung carcinoid (2%), prostate (2%), thyroid (1%), pancreas (1%), and other sites (3%) [8]. To our knowledge, this meta-analysis represents the largest systematic effort to report on esophageal cancer metastasis to the eye, synthesizing data from 41 individual cases in the literature. Since ocular metastasis from esophageal cancer is extremely rare, regular ophthalmologic screening in asymptomatic patients is not recommended. Current follow-up protocols after esophagectomy or chemoradiotherapy typically include endoscopic and radiologic evaluations focused on locoregional recurrence and distant metastases, but do not routinely assess ocular involvement [51]. However, in patients with symptoms such as blurred vision, visual field defects, eye pain, or redness, oncologists should maintain a high index of suspicion. Prompt referral to an ophthalmologist is critical for early recognition and timely intervention. Raising awareness among healthcare providers and patients regarding the potential for ocular involvement, albeit rare, may facilitate earlier detection. Multidisciplinary collaboration among oncologists, ophthalmologists, and surgeons is essential to ensure coordinated and effective management.

The prognosis of patients with ocular metastasis from esophageal cancer remains poor, reflecting the aggressive nature of the disease and its typically advanced stage at the time of diagnosis. In this meta-analysis, the median overall survival following ocular involvement was 6 months, with 1-year and 2-year survival rates of 23.7% and 9.5%, respectively. These outcomes are consistent with survival data for metastatic esophageal cancer involving more common sites. For example, patients with hepatic metastases have a median survival of approximately 5–6 months, while those with distant lymph node involvement may have slightly longer survival depending on disease burden and treatment response [52]. Although ocular metastasis may suggest a lower visceral tumor burden, our findings indicate that it typically coexists with systemic dissemination and does not confer a survival advantage over other metastatic presentations. Notably, in our cohort, 80.5% of patients had concurrent systemic metastases at the time of ocular involvement, reinforcing that ocular metastasis most often reflects widespread disease rather than isolated dissemination. Even in the subset of eight patients (19.5%), in whom ocular involvement was the sole site of documented metastatic disease at presentation, no significant survival advantage was observed compared with patients with multisite metastases.

Oligometastatic esophagogastric cancer is defined in the European clinical practice guidelines (OMEC-4) as a distinct biological and clinical state that may benefit from aggressive local treatment in selected patients [53]. However, the findings of our analysis suggest that ocular metastasis from esophageal cancer rarely occurs as an isolated manifestation and is most often associated with disseminated systemic disease. In our cohort, overall survival remained poor even for patients whose ocular lesion appeared to represent the only detectable metastatic site at the time of diagnosis. These observations suggest that ocular metastasis from esophageal cancer may not represent a classical oligometastatic phenotype, but more likely reflects advanced systemic disease. Nevertheless, given the limited number of reported cases and the methodological limitations of case reports, this should be interpreted with caution. Further multicenter studies and dedicated registries are required to establish whether a subset of patients with isolated ocular involvement could be considered and treated within the oligometastatic disease framework.

Despite the poor overall prognosis associated with ocular metastasis from esophageal cancer, local therapy remains an essential component of patient management because of the impact of ocular symptoms on quality of life [54]. Blurred vision, floaters, diplopia, eye pain, and photopsia were among the most common manifestations in our cohort, often leading to functional impairment. Therefore, local interventions are indicated not to prolong survival, but for symptomatic relief and preservation of visual function with minimal toxicity to offer a better quality of life [55]. In our meta-analysis, most patients received local treatment, generally in the form of EBRT, given to 68.3% of patients. The EBRT regimens varied and included both conventional fractionation and stereotactic body radiotherapy. Approximately two-thirds of the 12 patients with available outcome data experienced improvement in visual acuity or resolution of ocular symptoms, while stabilization of visual function was achieved in the remaining one-third. Selected patients were given adjunctive therapies such as intravitreal injections and surgical procedures, including vitrectomy or enucleation, for palliation or diagnostic clarification. Taken together, our findings strongly support the role of aggressive, eye-directed therapy for carefully selected patients, as such interventions can lead to meaningful improvement in vision and overall quality of life, even in the context of disseminated disease.

This meta-analysis is subject to several limitations, primarily stemming from the rarity of ocular metastasis from esophageal cancer. First, the low incidence of metastasis to this location limits the volume of available scientific literature, which consists exclusively of case reports and small case series. These reports inherently lack statistical power and external validity, thereby constraining the generalizability of our findings. Second, the possibility of publication bias cannot be excluded, as reported cases may disproportionately represent patients with severe symptoms, poor prognosis, or unusual clinical courses, potentially skewing survival outcomes. Third, although univariate statistical analysis was performed to explore associations between clinical variables and survival, no significant findings were identified. The lack of significant associations should be interpreted with caution and likely reflects the small sample size and limited statistical power rather than the absence of true prognostic effects. Additionally, there was marked heterogeneity in diagnostic modalities, therapeutic approaches, further complicating direct comparison of results introducing interpretative inconsistencies. Moreover, essential prognostic modifiers such as patient comorbidities, performance status, molecular profiles, and therapies received were inconsistently reported or entirely absent in the primary studies, precluding their inclusion in the analysis. Finally, follow-up duration varied substantially among the reported cases, ranging from a few weeks to several years. Notably, the wide 95% confidence interval for the 2-year survival estimate (0%–25%) suggests substantial right-censoring, likely reflecting the small number of patients with long-term follow-up. Consequently, survival estimates should be considered in the context of these limitations, as the small sample size and limited statistical power may obscure true prognostic associations and affect the reliability of conclusions about the long-term outcomes of patients with ocular metastasis from esophageal cancer. Despite these limitations, our study highlights a critical and under-recognized issue; namely, the occurrence of ocular metastases from esophageal cancer; and provides the most comprehensive overview to date. However, it should serve primarily to generate hypotheses and awareness rather than definitive practice-changing evidence.

Given the rarity of ocular metastasis from esophageal cancer, there is a clear need to establish an international, site-specific registry dedicated to these cases. A centralized database would allow for the systematic collection of detailed clinical, imaging, histopathological, molecular, and treatment data. Such an initiative would serve multiple critical purposes. First, it would support efforts to clarify the underlying mechanisms of ocular dissemination of esophageal disease, whether driven by hematogenous pathways, specific adhesion molecules, or microenvironmental factors that facilitate ocular tropism. Moreover, the accumulation of molecular profiling data could reveal alterations or biomarkers specific to ocular metastases, potentially guiding targeted therapy. The registry could also define whether ocular involvement should be considered within the framework of oligometastatic disease. This would have significant implications for treatment planning, including the possible role of curative-intent local therapy for selected patients.

The management of ocular metastasis from esophageal cancer necessitates close multidisciplinary collaboration among oncologists, ophthalmologists, radiation oncologists, and surgeons. Early recognition and timely intervention are critical, particularly given the rapid progression and distressing nature of ocular symptoms. Oncologists must maintain a high index of suspicion and promptly refer patients with new or unexplained visual disturbances. Ophthalmologists, in turn, should be equipped to recognize metastatic lesions and initiate appropriate diagnostic and therapeutic pathways. Surgeons and radiation oncologists also play pivotal roles in managing complex or refractory cases, particularly when enucleation or targeted radiotherapy is required. A coordinated team approach could help optimize patient quality of life even in the context of advanced systemic disease.

## Conclusions

Ocular metastasis from esophageal cancer is a rare but clinically significant manifestation, often indicating disseminated disease. Despite its poor overall prognosis, timely diagnosis and aggressive local treatment can improve visual outcomes and quality of life in selected patients. Further international collaboration and the development of a dedicated registry are essential to enhance our understanding and guide evidence-based management of this uncommon metastatic presentation.
